# Combined Stromal Vascular Fraction and HGF-Functionalized Self-Assembling Peptide Hydrogel Improves Intracerebral Hemorrhage Repair in Rats

**DOI:** 10.3390/gels12030257

**Published:** 2026-03-19

**Authors:** Xuhuai Chen, Tiantian Li, Feng Yang, Yanling Chen, Yuanyi Liu, Linshu Ding, Jialin Li, Haibo Zhou, Qiuju Yuan, Wutian Wu

**Affiliations:** 1 Guangdong-Hong Kong-Macau Institute of CNS Regeneration, College of Life Science and Technology, Jinan University, Guangzhou 510632, China; 2 Re-Stem Biotechnology Co., Ltd., Suzhou 215129, China

**Keywords:** intracerebral hemorrhage, self-assembling peptide hydrogel, stromal vascular fraction, neuroinflammation, neuroprotection

## Abstract

Intracerebral hemorrhage (ICH) remains a devastating condition with no available therapies that can effectively mitigate secondary injury and promote neurological repair. This research presents a novel combinatorial regenerative strategy, concurrently delivering adipose-derived stromal vascular fraction (SVF) within an adhesive self-assembling peptide (HGF-RADA16-IKVAV) nanohydrogel (HGF). In a clinically relevant rat model of ICH with hematoma evacuation, the combined therapy of HGF and SVF demonstrated synergistic and enhanced efficacy. In the short term, the combined therapy demonstrated hemostatic benefits, and significantly reduced hematoma volume, brain edema, neuronal apoptosis and neuroinflammation indicated by pro-inflammatory markers (NLRP3, caspase-1, Iba-1, CD68, GFAP) while increasing the levels of anti-inflammatory (CD206) and angiogenic (CD31) markers. Longitudinal behavioral assessments conducted over six weeks demonstrated persistent and significant improvements in motor coordination, forelimb strength, and gait parameters within the HGF + SVF group, surpassing all monotherapies. Ultrastructural analysis also showed that myelinated axons were better preserved at the injury border, with thicker myelin sheaths. These findings demonstrate that the co-administration of SVF with an adhesive and hemostatic hydrogel collaboratively diminishes secondary injury, modulates neuroinflammation, and promotes functional and structural brain recovery following ICH, indicating a promising and translatable strategy.

## 1. Introduction

Intracerebral hemorrhage (ICH), accounting for 10–20% of all strokes, remains a catastrophic neurological event marked by persistently high mortality and extended disability rates, with no efficacious treatments available to promote recovery [1,2]. The initial hematoma causes the initial mechanical injury, and the outcome is mostly determined by the secondary injury cascade, a complex series of events that unfold over hours to weeks [3,4]. This cascade involves harmful blood breakdown products (heme, iron) that cause oxidative stress, damage to the blood–brain barrier that leads to pronounced edema, and a strong, often long-lasting inflammatory condition exacerbates neuronal apoptosis and axonal injury, ultimately resulting in the development of a cystic cavity surrounded by a glial scar, which hinders endogenous recovery [3,4,5]. Present management focuses on hematoma evacuation and supportive care, which do not modify the fundamental destructive pathophysiology, thus requiring interventions that can mitigate secondary injury and enhance regeneration [6]. In the clinical setting of minimally invasive hematoma evacuation, direct access to the bleeding vessel for traditional hemostasis is often limited, and re-bleeding from the cavity wall remains a concern. Therefore, an injectable biomaterial that concurrently provides hemostatic sealing and structural support within the evacuated cavity offers a distinct therapeutic advantage [1,7].

In this situation, strategies that combine regenerative biomaterials and cell therapy appear promising for addressing multiple problems at once. Injectable biomaterial scaffolds can be placed in the empty lesion cavity to give it structural support and change the local microenvironment [8,9]. Synthetic self-assembling peptide hydrogels are beneficial due to their biocompatibility, nanofibrous architecture that emulates the extracellular matrix, and suitability for minimally invasive administration [10,11]. They can reduce cavitation and furnish a substrate conducive to repair mechanisms [12]. In this study, we used a functionalized peptide hydrogel called HGF-RADA16-IKVAV. The term ‘HGF’ refers to a tripeptide motif (HisGlyPhe) that we previously showed is able to enhance the hydrogel’s tissue adhesiveness and injectability, facilitating surgical handling and stability within the lesion cavity [13]. Importantly, self-assembling peptide hydrogels themselves have been reported to exhibit hemostatic properties in preclinical bleeding models [7,14], an attribute directly relevant to ICH where rapid stabilization of the bleeding site is critical. This ability is directly related to ICH because one of the main goals of treatment is to quickly stabilise the bleeding site to stop the growth of the hematoma. Pioneering studies have established self-assembling peptide hydrogels (e.g., RADA16) as promising scaffolds for neural regeneration due to their ECM-mimicking nanofiber structure and biocompatibility [14,15]. These hydrogels also exhibit inherent hemostatic properties in preclinical bleeding models [7]. To enhance functionality, bioactive motifs can be incorporated: the IKVAV sequence promotes neurite outgrowth and neuronal adhesion [16], while the HGF (His-Gly-Phe) motif identified in our prior work improves tissue adhesiveness and injectability [13]. Although peptide hydrogels have been combined with stem cells for CNS repair [17], the combination of a hemostatic, adhesive hydrogel with SVF for ICH remains unexplored, supporting the novelty of our approach.

Along with improvements in biomaterials, cell-based therapies have shown great promise for treating the complex problems associated with CNS injuries. Mesenchymal stem/stromal cells (MSCs), recognized for their immunomodulatory and trophic paracrine roles, have been the subject of extensive research [18,19,20,21,22]. However, their clinical utilization is constrained by prolonged ex vivo expansion, the risk of phenotypic drift, and complex regulatory frameworks [23]. The adipose-derived SVF presents a compelling alternative as an easily accessible, autologous cellular product acquired with minimal processing [24,25,26,27]. SVF is a mixture of different types of cells, such as stem/stromal cells, endothelial progenitor cells, and immunomodulatory cells [25,27]. This diversity enables a multifaceted therapeutic effect: SVF releases a combination of angiogenic (e.g., VEGF), neurotrophic (e.g., BDNF), and multiple regenerative effects [28,29,30,31,32]. Its application in cerebral hemorrhage has yet to be investigated.

A major problem with intracerebral cell therapy is that the cells do not survive long because the environment is very inflammatory and cytotoxic at first [32]. We propose that delivering SVF in conjunction with a supportive biomaterial can overcome this limitation through a sequential and synergistic mechanism. This combinatorial strategy operates in two distinct stages. First, the HGF-functionalized self-assembling peptide hydrogel provides immediate hemostasis and physical stabilization of the lesion cavity, thereby reducing early hematoma expansion and mitigating secondary injury cascades [13]. Second, the hydrogel serves as a protective, biocompatible scaffold that enhances SVF cell retention and survival within the harsh ICH microenvironment, shielding transplanted cells from cytotoxic and inflammatory insults [33,34]. Within this stable niche, the engrafted SVF is hypothesized to exert sustained paracrine effects—including immunomodulation, angiogenesis, and neuroprotection—which are further amplified by the supportive hydrogel matrix [35]. This integrated two-stage therapeutic logic culminates in enhanced functional recovery following ICH.

Consequently, this study constitutes the first examination of the therapeutic efficacy of SVF, both in isolation and in combination with the functionalized HGF-RADA16-IKVAV hydrogel, within a clinically relevant rat model of ICH characterized by partial hematoma evacuation. We hypothesize that this combined approach will yield superior outcomes by leveraging the hydrogel’s acute-phase mechanisms—hemostasis and structural stabilization—and SVF’s sustained reparative functions, including immunomodulation and tissue repair. This synergistic, two-stage strategy is expected to more effectively mitigate secondary injury, enhance neuroprotection and angiogenesis, and ultimately promote long-term functional and structural recovery.

## 2. Results and Discussion

1. Long-term retention of transplanted SVF cells in the injured brain

**Figure 1:**
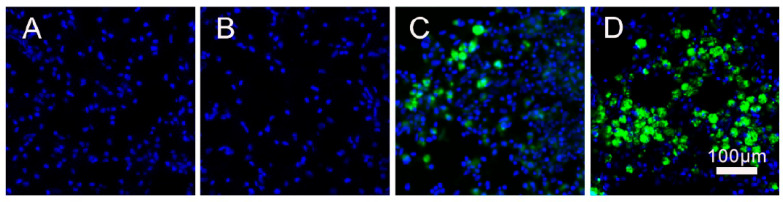
Cell retention at 6 weeks after transplantation of SVF and HGF + SVF (*n* = 6 per group). Representative fluorescence images of coronal cryosections through the center of the lesion cavity are shown. GFP^+^ SVF cells (green) appear in the SVF group (**C**) and the HGF + SVF group (**D**), localized within the lesion boundary. DAPI (blue) stains nuclei. (**A**): Saline group. (**B**): HGF group. (**C**): SVF group. (**D**): SVF + HGF group. Scale bar = 100 µm.

To examine whether there was adequate cell engraftment following ICH, we monitored green fluorescent protein (GFP)-labeled SVF cells. No GFP+ signal was observed in saline (Figure 1A) and HGF groups (Figure 1B). Six weeks after the transplant, a strong GFP+ signal was found in the HGF + SVF group, mostly in the tissue around the resolved injury cavity (Figure 1D). Conversely, only sparse, minimal GFP+ signals were detected in the SVF-only group (Figure 1C), suggesting accelerated cell clearance. This discovery substantiates that the RADA16-IKVAV hydrogel functions as an essential scaffold, offering a protective physical niche for SVF cells against the adverse microenvironment and promoting their extended retention at the lesion site. Notably, because the SVF was used as an unfractionated mixture, the specific cell type(s) responsible for long-term survival remain to be identified.

To further assess the global distribution of engrafted SVF cells, we systematically examined all serial coronal sections covering the entire lesion area (from bregma +1.5 mm to −2.5 mm) in each animal. In the HGF + SVF group, GFP^+^ signals were consistently and exclusively confined to the lesion cavity and its immediate periphery, with no detectable fluorescence in the contralateral hemisphere or in ipsilateral regions distant from the injury site. This spatially restricted pattern was observed in every animal of the combination group, strongly indicating that the HGF hydrogel effectively retains SVF cells locally and prevents their widespread migration. These observations provide critical qualitative evidence that the combinatorial approach successfully establishes a localized cellular niche for sustained paracrine therapeutic effects.

2. The HGF + SVF Combination Most Effectively Mitigates Acute Hematoma Expansion and Brain Edema

**Figure 2:**
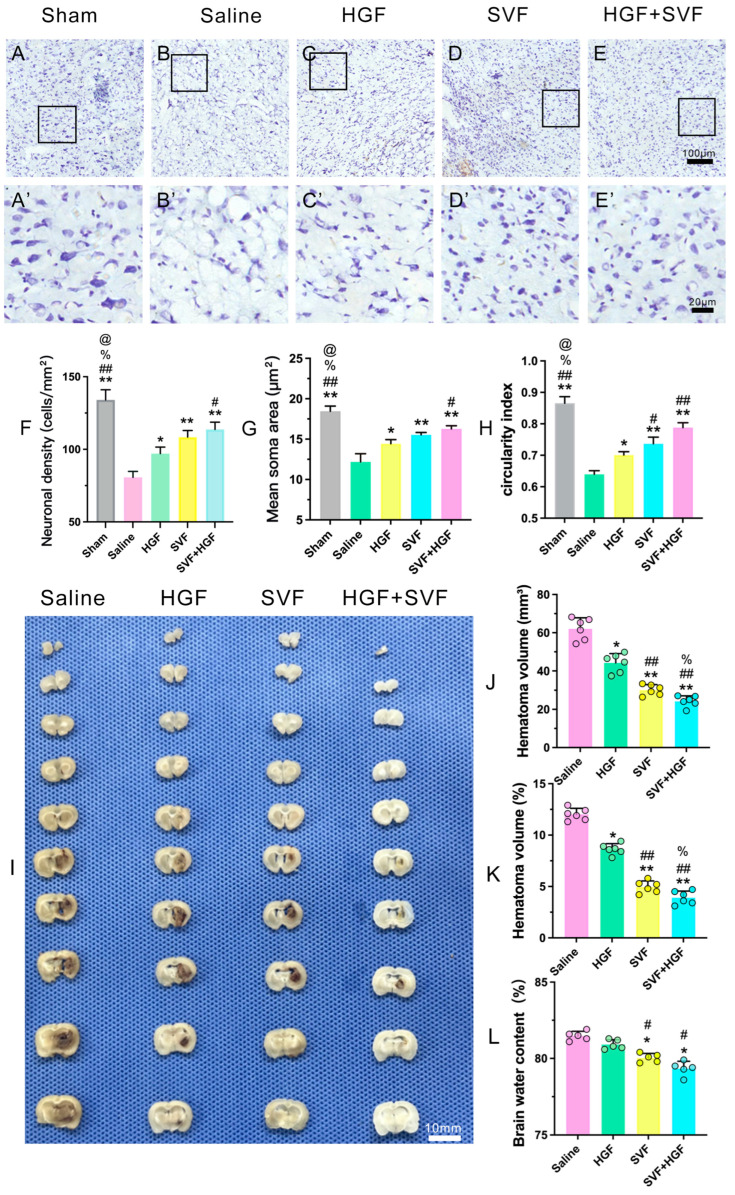
(**A**–**E**) Representative Nissl staining images of the peri-hematomal region at 3 days after ICH. (**A’**–**E’**) are higher magnification images of the insets in (**A**), (**B**), (**C**), (**D**) and (**E**), respectively. (**F**) Quantitative analysis of neuronal density (cells/mm^2^). (**G**) Quantitative analysis of mean soma area (μm^2^). (**H**) Quantitative analysis of circularity index. Data are presented as mean ± SD (*n* = 6 per group). (**I**) Hematoma size of each group at 3 days after ICH. Scale bar in (**E**) = 100 µm. Scale bar in (**E’**) = 20 µm. Scale bar in (**I**) = 10 mm (*n* = 6). (**J**) Quantitative analysis of the absolute hematoma volume of each group. (**K**) Quantitative analysis of the relative hematoma volume of each group. (**L**) Quantitative analysis of water content in the ipsilateral cerebral tissue of each group at 3 days after ICH (*n* = 5). * *p* < 0.05 versus the saline group. ** *p* < 0.01 versus the saline group. # *p* < 0.05 versus the HGF group. ## *p* < 0.01 versus the HGF group. % *p* < 0.05 versus the SVF group. @ *p* < 0.05 versus the HGF + SVF group.

Evaluation of acute brain injury at 3 dpi demonstrated substantial therapeutic effects. Nissl staining revealed significant neuronal loss, pyknosis, and vacuolization in the ipsilateral striatum of the saline group (Figure 2B,B’), which was markedly diminished in all treatment groups (Figure 2C–E,C’–E’). Quantitative morphological analysis of Nissl-stained neurons in the peri-hematomal region further substantiated the neuroprotective effect of the combined therapy (Figure 2F–H). The HGF + SVF group exhibited the highest neuronal density (Figure 2F), significantly exceeding that of the saline, HGF, and SVF groups. Moreover, neurons in the HGF + SVF group displayed a significantly larger mean soma area compared to all other groups (Figure 2G), indicating better preservation of neuronal size or healthier neuronal status. Importantly, the circularity index, reflecting neuronal shape integrity, was significantly higher in the HGF + SVF group than in the saline and monotherapy groups (Figure 2H), suggesting that the surviving neurons retained a more normal, rounded morphology rather than becoming shrunken or distorted. These results collectively demonstrate that HGF + SVF treatment not only preserves neuronal numbers but also maintains the structural integrity of surviving neurons in the peri-hematomal region. Quantitative analysis indicated that the combined HGF + SVF therapy exerted the most significant effect on primary injury markers. On day 3 after ICH, hematoma size and brain water content were assessed. Compared to the saline group, all treatment groups showed a significant reduction in the absolute hematoma volume and relative hematoma volume (Figure 2I–K). The most pronounced reduction occurred in the HGF + SVF group (Figure 2I–K). Similarly, brain water content was significantly lower in the treatment groups than in the saline group (Figure 2L). Again, the strongest reduction was observed in the combined HGF + SVF group (Figure 2L).

**Figure 3:**
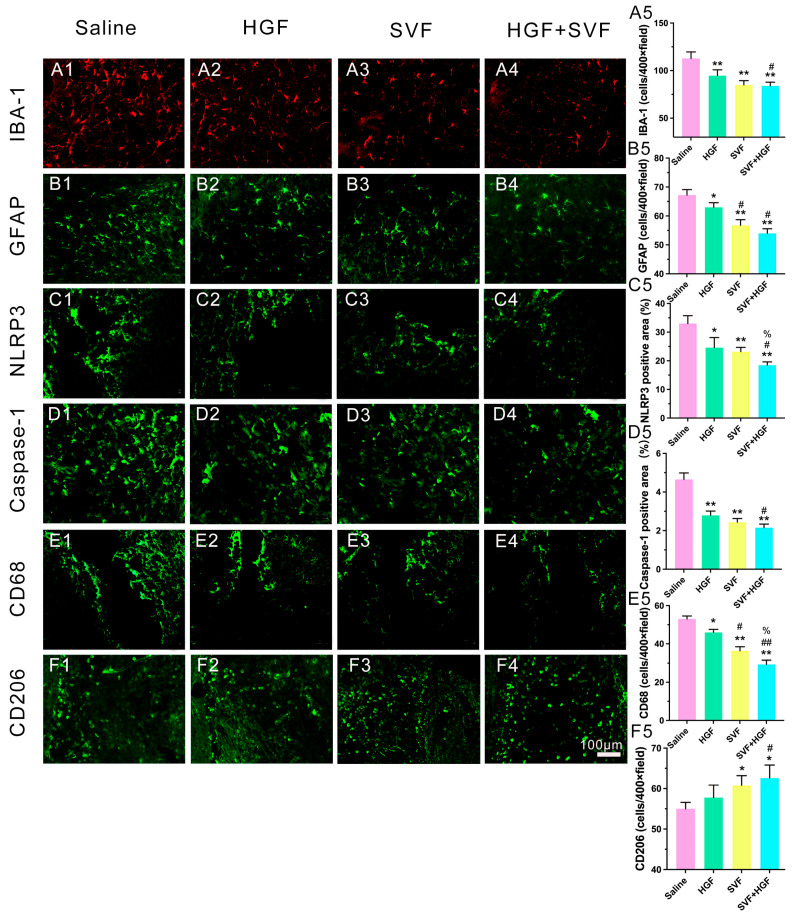
Effects of HGF, SVF, and HGF + SVF on peri-hematomal cells. (**A1**–**F4**) Representative immunofluorescence staining images for IBA-1, GFAP, NLRP3, caspase-1, CD68 and CD206 in the peri-hematomal region at day 3 after ICH in each group. Scale bar = 100 μm. (**A5**–**F5**) Quantitative analysis of IBA-1-positive cells, GFAP-positive cells, NLRP3-positive area, caspase-1-positive area, CD68-positive cells and CD206-positive cells in each group. Data are presented as mean ± SD (*n* = 6). * *p* < 0.05 versus the saline group. ** *p* < 0.01 versus the saline group. # *p* < 0.05 versus the HGF group. ## *p* < 0.01 versus the HGF group. % *p* < 0.05 versus the SVF group.

3. Synergistic modulation of neuroinflammation and facilitation of early repair mechanisms.
4. Inhibition of Pro-inflammatory Activation: The peri-hematomal region in the saline group was densely populated with activated Iba-1+ microglia (Figure 3A1), hypertrophic GFAP+ astrocytes (Figure 3B1), and CD68+ phagocytic microglia/macrophages (Figure 3E1). The levels of the inflammasome components NLRP3 and cleaved caspase-1 were high (Figure 3C1 and Figure 3D1, respectively). All treatments diminished these markers (Figure 3), with the HGF + SVF combination exhibiting the most significant suppression (Figure 3).

Promotion of an Anti-inflammatory Phenotype: In contrast, the HGF + SVF group exhibited a significantly increased area of CD206^+^ signal, indicating an elevated presence of cells with an anti-inflammatory/repair-associated phenotype (Figure 3F4).

**Figure 4:**
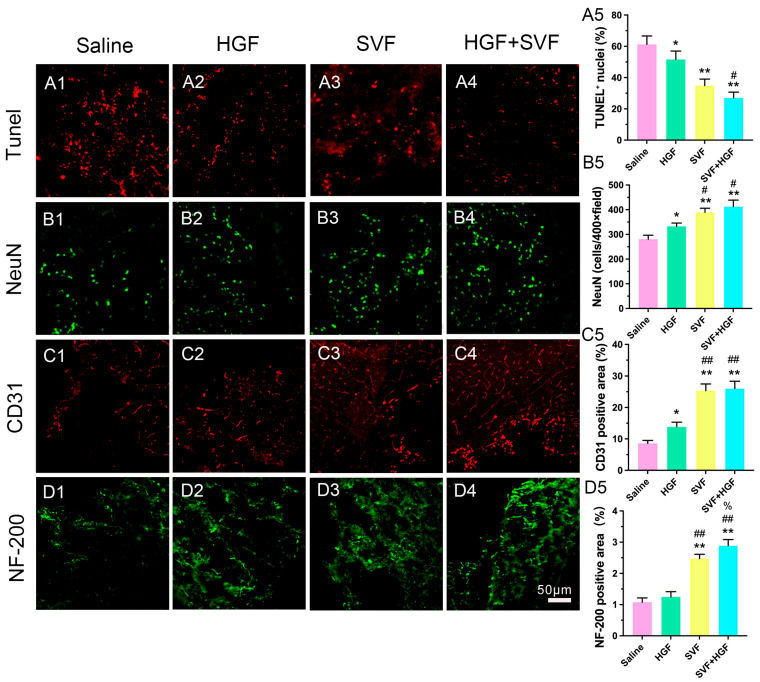
Effects of HGF, SVF, and HGF + SVF on Peri-hematomal Cells. (**A1**–**D4**) Representative immunofluorescence staining images for TUNEL, NeuN, CD31, and NF-200 in the peri-hematomal region at day 3 after ICH in each group. Scale bar = 100 µm. (**A5**–**D5**) Quantitative analysis of the percentage of TUNEL^+^ nuclei, NeuN-positive cells, CD31-positive area, and NF-200-positive area in each group. Data are presented as mean ± SD (*n* = 6). * *p* < 0.05 versus the saline group. ** *p* < 0.01 versus the saline group. # *p* < 0.05 versus the HGF group. ## *p* < 0.01 versus the HGF group. % *p* < 0.05 versus the SVF group.

Improved neuroprotection, angiogenesis, and axonal preservation: TUNEL+ apoptotic cells were prevalent in the saline group (Figure 4A1,A5) but significantly diminished in the HGF + SVF group (Figure 4A4,A5). NeuN+ neuronal survival in the peri-hematomal penumbra was consistently highest in the HGF + SVF group (Figure 4B4,B5). Additionally, this group demonstrated a notable rise in CD31+ microvessel density, indicating augmented angiogenesis (Figure 4C4,C5), and an increased preservation of NF-200+ neurofilaments, signifying axonal protection (Figure 4D4,D5).

5. Long-lasting and better functional neurological recovery

**Figure 5:**
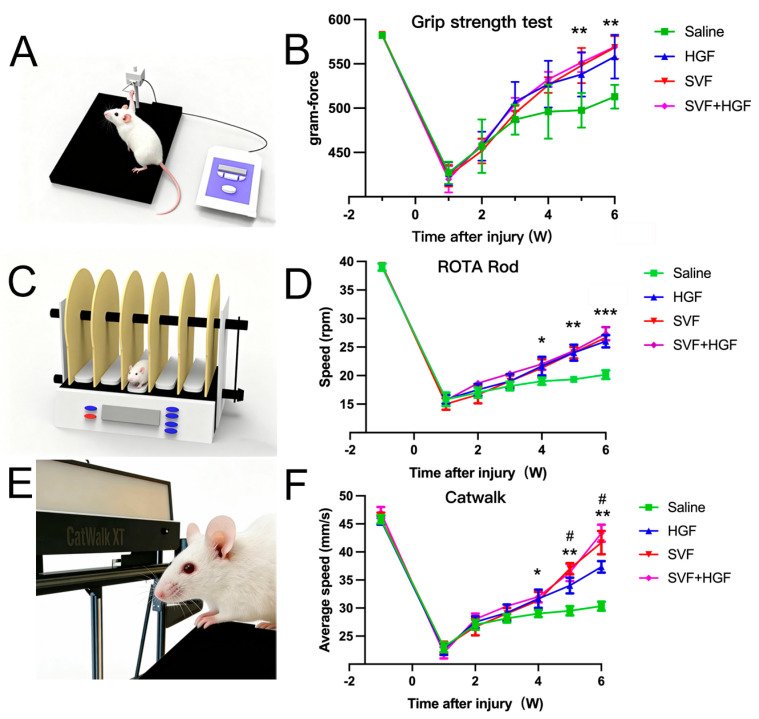
(**A**,**B**) Grip strength test results. (**C**,**D**) Rotarod test results. (**E**,**F**) Catwalk results; behavioral assessments were performed weekly until 6 weeks after ICH in each group. Data are presented as the mean ± SD (*n* = 6). * *p* < 0.05 versus the saline group. ** *p* < 0.01 versus the saline group. *** *p* < 0.001 versus the saline group. # *p* < 0.05 versus the HGF group.

Longitudinal behavioral testing conducted over six weeks identified a clear hierarchy in therapeutic efficacy, with the HGF + SVF combination group exhibiting the most pronounced and enduring functional neurological recovery across various domains (Figure 5). The HGF + SVF treatment restored more than 90% of the baseline grip strength in the forelimb, which was significantly better than the monotherapy groups (Figure 5A,B). This group made almost a full recovery on the accelerating rotarod by week six in terms of motor coordination and endurance (Figure 5C,D). They performed significantly better than all the other groups (Figure 5C,D). Additionally, sensitive CatWalk gait analysis revealed that the HGF + SVF cohort demonstrated the most significant enhancement in the Maximum Contact Area and Swing Speed of the affected forepaw, as well as the highest Regularity Index (Figure 5E,F), signifying the most coordinated and symmetrical locomotor function.

6. Improved ultrastructural repair and myelination at the lesion border

**Figure 6:**
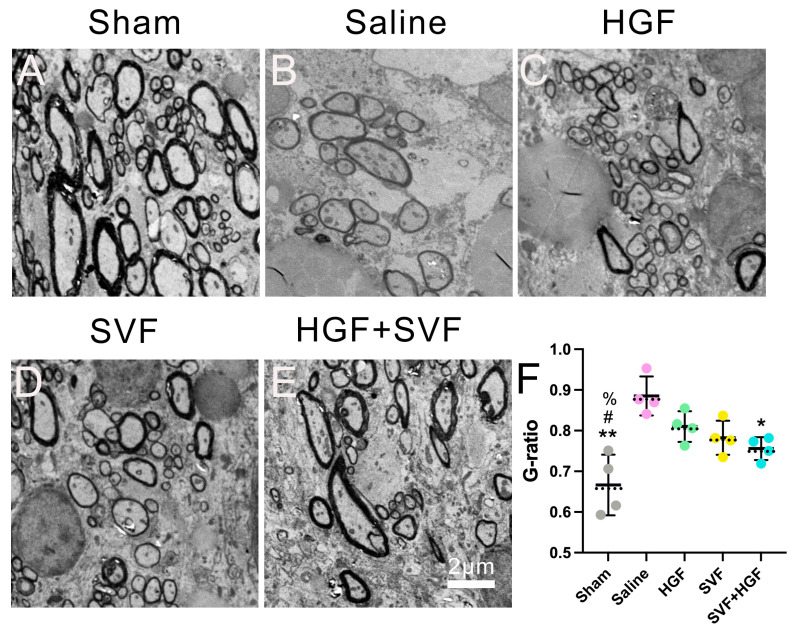
Representative electron microscopy images and quantification of the G-ratio. Samples were collected 6 weeks post-treatment in sham (**A**), saline (**B**), HGF (**C**), SVF (**D**) and HGF+SVF (**E**) groups. Data are presented as raw data points with mean ± SD (solid line) and median (dashed line) indicated (*n* = 4). * *p* < 0.05 versus the saline group. ** *p* < 0.01 versus the saline group. # *p* < 0.05 versus the HGF group. % *p* < 0.05 versus the SVF group. Scale bar = 2 μm.

Transmission electron microscopy of the lesion border zone at six weeks yielded substantial evidence of structural repair. In the sham group, normal axons were consistently encased by intact myelin sheaths (Figure 6A). In contrast, the saline group showed a disorganized neuropil with few, thinly myelinated axons and clear signs of axonal degeneration (Figure 6B). The groups that received monotherapy showed some improvement. The HGF (Figure 6C) and SVF (Figure 6D) groups exhibited a higher quantity of myelinated axons with a more structured architecture compared to the saline group, although the myelin sheaths remained relatively thin. The HGF + SVF combination group, on the other hand, had a much higher density of myelinated axons with myelin sheaths that were much thicker and more compact (Figure 6E). Quantitative G-ratio analysis validated these morphological findings: the HGF + SVF group exhibited a significantly reduced mean G-ratio (0.756 ± 0.028) compared to the saline group (0.885 ± 0.048, *p* < 0.01) (Figure 6F). The median values were 0.761 (HGF + SVF) and 0.874 (saline), further confirming the consistency of the effect. This decrease suggests improved myelin preservation and/or active remyelination after combination therapy.

7. Discussion

This study presents the first evidence that the simultaneous local administration of autologous adipose-derived stromal vascular fraction (SVF) and a hemostatic, self-assembling peptide hydrogel (HGF-RADA16-IKVAV) synergistically improves histological, functional, and structural recovery in a preclinical model of intracerebral hemorrhage (ICH) with partial hematoma evacuation. A clear order of treatments was observed: HGF and SVF monotherapies were both more effective than the control, but the HGF + SVF combination always had the strongest restorative effects on almost all the measured parameters. This highlights the imperative for a combinatorial approach that sequentially addresses the complex pathophysiology of ICH.

The combined therapy is more effective due to its multi-stage mechanism. The foundation of this approach is rapid and reliable hemostasis, a critical but often overlooked component of effective acute ICH intervention. Our data demonstrate that the HGF hydrogel exhibits rapid hemostatic capacity in a cortical vessel transection model (Figure S1), achieving complete hemostasis in approximately 4 s, which is much faster than the 65 s seen in the control group. It should be noted, however, that this was not directly quantified within the ICH cavity; therefore, the hemostatic contribution in the ICH model is inferred from hematoma volume reduction and should be interpreted with caution. This immediate gelation and sealing of the lesion cavity directly address the primary instability of ruptured vasculature. The stable nanofibrous network that forms acts as both a physical sealant and a clot stabilizer. This lowers the risk of re-bleeding and hematoma expansion. The most significant decrease in hematoma volume and peri-hematomal edema on day 3 in the combination group is a direct result of this strong early physical stabilization. This treatment stops the main mass effect and slows down the first release of neurotoxic blood breakdown products (like heme and iron). This may help interrupt the secondary injury cascade at an early stage. The observation that SVF alone significantly reduced hematoma volume (Figure 2G) suggests that the cellular component may contribute to hemostasis and early hematoma resolution through mechanisms distinct from the hydrogel’s physical sealing. Possible explanations include the release of procoagulant factors and promotion of vascular repair [36,37], although these mechanisms were not directly assessed in the present study. The lack of a significant additive effect of HGF and SVF on hematoma volume may reflect a ceiling effect or the fact that the two components act on different timescales, with the hydrogel providing immediate stabilization and SVF exerting sustained reparative effects. Indeed, the synergistic benefit of the combination was most evident in long-term functional recovery and structural preservation (Figure 5 and Figure 6).

The cellular composition of SVF is a critical determinant of its therapeutic potential. In this study, we combined flow cytometry and single-cell RNA sequencing to provide, to our knowledge, the first single-cell resolution map of rat SVF. Flow cytometry revealed the heterogeneity of freshly isolated SVF, with 3.49% of cells expressing the endothelial marker CD31, 38.35% expressing the hematopoietic marker CD45, and 50.56% expressing the mesenchymal stromal cell marker CD90 (Supplementary Figure S2). More importantly, single-cell transcriptome analysis uncovered an unexpectedly rich cellular heterogeneity, with SVF comprising 11 distinct subtypes, including macrophages (28.01%), T cells (20.01%), adipose stem and progenitor cells (ASPCs, 16.74%), and endothelial cells (5.91%), among others (Supplementary Figure S3; Supplementary Table S1). This diversity mirrors the complexity of human SVF and suggests that the therapeutic effects observed in our ICH model—including immunomodulation, angiogenesis, and neuroprotection—may arise from the coordinated actions of multiple cell types. However, it should be emphasized that the precise contribution of individual subpopulations to these functional outcomes remains to be determined, and the associations proposed here are speculative at this stage.

Beyond characterizing the cells, we also demonstrated that the HGF-functionalized hydrogel provides a supportive niche for SVF. In vitro 3D culture showed that GFP-labeled SVF cells adhered to the hydrogel matrix, maintained their morphology, and extended cellular protrusions over 8 days (Supplementary Figure S4), indicating active interaction with the IKVAV and HGF motifs. These findings are consistent with the known properties of RADA16-based peptides in promoting cell adhesion and survival, and they mechanistically support the enhanced cell retention observed in vivo (Figure 1D). Collectively, these data establish that the HGF hydrogel not only serves as a physical scaffold but also actively supports the viability and function of embedded SVF cells, thereby maximizing their therapeutic output.

Following this initial stabilization, the therapy is hypothesized to enter a second phase now that this base is stable. The hydrogel matrix acts as a three-dimensional scaffold that protects the retained SVF. This diverse group of cells, which includes stromal, endothelial progenitor, and immunomodulatory cells, then carries out a wide range of paracrine activities. The significant decrease in apoptotic cells (TUNEL+) and enhanced neuronal preservation (NeuN+) in the combination group are likely facilitated by a localized “trophic shield” of neurotrophic factors (e.g., BDNF, NGF) secreted by the SVF, which improves neuronal resilience.

A defining feature of the combined therapy was its coordinated alteration of the post-ICH neuroimmune environment. This regimen most effectively suppressed the activation of microglia (Iba-1+) and astrocytes (GFAP+), and reduced the immunoreactivity of NLRP3 and cleaved caspase-1. The increase in CD206 immunoreactivity, together with reduced CD68 signal, suggests a shift in the local immune environment toward a pro-reparative state, a transition potentially influenced by SVF-derived mediators [28,29,30,31]. However, because we did not perform double-labeling for CD68 and CD206, we cannot definitively conclude that individual cells underwent classical M1-to-M2 phenotypic switching. The specific cellular source(s) of these mediators could not be identified in the present study due to the lack of phenotypic analysis. This remains an important limitation.

The substantial rise in CD31+ microvessel density in the combination group indicates effective angiogenesis [38,39], supported by SVF-derived pro-angiogenic factors and the accommodating structural framework of the hydrogel matrix. This neovascularization improves local blood flow and may also facilitate the clearance of waste products.

The longitudinal behavioral outcomes over six weeks provide robust translational evidence. The combination group demonstrated enhanced and prolonged recovery in motor coordination (rotarod), strength (grip test), and gait symmetry/coordination (CatWalk), nearing normal functionality. This indicates that the therapy not only safeguarded tissue but also promoted adaptive neuroplasticity and the restoration of functional sensorimotor circuits.

Ultrastructural examination using transmission electron microscopy demonstrated the therapy’s ability to facilitate microstructural repair. The combination group exhibited a diminished G-ratio (signifying thicker myelin sheaths) and an increased density of preserved myelinated axons at the lesion periphery. This remyelination may be attributed to several potential mechanisms, including: (1) oligodendrocytes are directly protected; (2) SVF-derived factors (like PDGF-AA and IGF-1) stimulate oligodendrocyte precursor cells [40,41,42,43,44]; and (3) the combined therapy creates a pro-regenerative microenvironment. This transition from neuroprotection to proactive facilitation of network repair signifies a therapeutic progression.

8. Limitations and Future Directions

This study has several limitations. First, although the hydrogel’s hemostatic efficacy was quantitatively demonstrated in a cortical vessel model, its direct effect on bleeding kinetics within the ICH cavity remains to be verified by intravital imaging techniques in future studies. Second, we did not perform phenotypic characterization of the transplanted SVF by flow cytometry. Although the SVF was isolated using a standardized, collagenase-based protocol widely used in preclinical and clinical studies [45], its cellular composition—particularly the proportions of CD90^+^ mesenchymal stromal cells, CD31^+^ endothelial progenitors, and CD45^+^ hematopoietic cells—was not quantified. This omission has two important implications: (i) it limits our ability to correlate therapeutic efficacy with specific SVF subpopulations, and (ii) it reduces the batch-to-batch reproducibility of the cell product. Consequently, our mechanistic interpretations regarding angiogenesis, immunomodulation, and neuroprotection are based on the collective activity of the whole SVF, not on isolated subsets. Future studies should incorporate routine flow cytometric quality control of each SVF preparation, and more advanced techniques such as single-cell RNA sequencing should be employed to deconvolute the cellular and molecular mechanisms underlying the therapeutic effects of SVF in ICH. Third, although our histological analysis focused on high-resolution imaging of the peri-hematomal region to quantitatively assess local cell retention, we did not perform dedicated whole-brain or hemisphere fluorescence imaging to map the three-dimensional distribution of engrafted SVF cells. We acknowledge that systematic whole-brain imaging (e.g., optical clearing, 3D reconstruction) would provide more definitive evidence of cell localization and migratory behavior. Nevertheless, our comprehensive examination of serial coronal sections spanning the entire lesion area consistently revealed that GFP^+^ cells were strictly confined to the lesion cavity and its immediate boundary, with no evidence of distant migration. This spatial confinement strongly corroborates the role of the HGF hydrogel as an effective local delivery and retention scaffold. Future studies employing advanced imaging techniques—such as tissue clearing and light-sheet microscopy—are warranted to fully elucidate the spatiotemporal dynamics of SVF cells within the injured brain, particularly in larger animal models with gyrencephalic brains that more closely resemble human neuroanatomy. Fourth, translation to clinical application requires testing in large animal models with gyrencephalic brains (e.g., pigs) to better recapitulate human neuroanatomy, cerebral blood flow dynamics, and surgical feasibility at a clinically relevant scale. Fifth, this study did not include in vitro characterization of SVF cell behavior (e.g., adhesion, proliferation, survival) within the HGF hydrogel. Such studies would provide valuable insights into the direct effects of the IKVAV and HGF motifs on cell function and help optimize the material design. Future work should systematically evaluate these parameters using 2D and 3D culture systems. Sixth, while the 2% HGF hydrogel formulation was adopted from our previously published and extensively characterized material, we did not perform independent rheometric validation for each experimental batch in this study. Although the hydrogel performed consistently in vivo (e.g., immediate gelation, effective hemostasis, and robust cell retention), future preclinical studies should incorporate routine batch-wise rheological quality control to further strengthen reproducibility and translational readiness. Seventh, the combination treatment was associated with decreased NLRP3 and cleaved caspase-1 signals in the peri-hematomal region, suggesting a possible modulation of inflammasome-related pathways. Definitive evidence of NLRP3 inflammasome inhibition would require additional biochemical validation, such as Western blotting and IL-1β/IL-18 quantification, which is a limitation of the current study. Eighth, this study did not investigate dose–response relationships or varying treatment initiation windows. Future studies should systematically evaluate these parameters to define optimal dosing and therapeutic time windows.

## 3. Conclusions

This study presents a logical and innovative combinatorial regenerative approach for ICH, integrating clinically available SVF with a sophisticated hemostatic-adhesive biomaterial (HGF). The system works along a “therapeutic continuum,” starting with instantaneous hemostasis, moving on to active immunomodulation and neuroprotection to reduce secondary injury, and ending with the promotion of angiogenesis, remyelination, and functional rewiring. The consistent synergistic advantages across all outcome measures strongly support an integrated strategy to address the intricate challenges of hemorrhagic brain injury. The HGF + SVF platform is a promising candidate for a full ICH treatment that can be used in other settings.

## 4. Materials and Methods

### 4.1. Animals

All animal experiments were approved by the Laboratory Animal Ethics Committee at Jinan University, China (Approval No. IACUC-20251029-06). Male Sprague Dawley (SD) rats (8 weeks old, 300 ± 20 g) were provided by Guangdong Medical Experimental Animal Center (Guangzhou, China; license no. SYXK (Yue) 2022-0174). For SVF isolation, GFP-transgenic Sprague-Dawley rats (on the same genetic background) served as donors to enable cell tracking. Animals were housed under a 12/12-h light/dark cycle with ad libitum access to food and water.

### 4.2. Preparation and Characterization of HGF-RADA-IKVAV Hydrogel

The HGF-RADA16-IKVAV (2%) hydrogel was prepared by dissolving the peptide in sterile double-distilled water. The peptide (sequence: H_2_N–RADA16–IKVAV–GG–HGF–CONH_2_; where HGF = His-Gly-Phe) was custom-synthesized by ChinaPeptides Co., Ltd. (Shanghai, China) with an N-terminal amide. Peptide purity (>95%) was confirmed by HPLC, and the molecular weight was verified by mass spectrometry (matched the expected value). The solution was filter-sterilized using a 0.22 μm syringe filter, and the pH was adjusted to 7.4, leading to rapid self-assembly of a stable, nanofibrous hydrogel within 30 min at room temperature. The solution was filter-sterilized using a 0.22 μm syringe filter, and the pH was adjusted to 7.4, leading to rapid self-assembly of a stable, nanofibrous hydrogel within 30 min at room temperature. The mechanical properties and nanofibrous structure of the hydrogel have been confirmed using rheometry and scanning electron microscopy, respectively, in our previous studies [13]. The peptide preparation and gelation protocols were followed in the current study; therefore, detailed rheological data are not repeated here.

### 4.3. Preparation of Adipose-Derived Stromal Vascular Fraction (SVF)

SVF was extracted from the bilateral inguinal adipose tissue pads of each GFP-transgenic rat immediately before the induction of ICH. GFP transgenic Sprague Dawley rats, which ubiquitously express EGFP under the CAG promoter, were used as donors for SVF isolation to enable long-term in vivo cell tracking. We cut up the fat into small pieces and mixed it with 0.1% collagenase type I (Worthington, Lakewood, NJ, USA) in PBS at 37 °C for 60 min, stirring gently. We filtered the digest through a 100-µm cell strainer to get rid of any debris and then spun it at 600× *g* for 5 min. The pellet, which had the SVF in it, was resuspended in erythrocyte lysis buffer for 5 min, washed twice with PBS, and then passed through a 40-µm strainer. Trypan blue (Sigma-Aldrich, St. Louis, MO, USA) exclusion was used to check cell viability, and the concentration was adjusted to 4 × 10^7^ cells/mL. SVF was used immediately after isolation as a heterogeneous, unfractionated cell population to preserve its native composition and maximize translational relevance. 

### 4.4. Intracerebral Hemorrhage (ICH) Model

The ICH model was induced by stereotactic injection of collagenase type IV into the striatum, as previously described [46,47]. The rats were anesthetized with an intraperitoneal injection of 1% pentobarbital sodium (40 mg/kg) and then put in a stereotactic frame (RWD Life Science, Shenzhen, China). A midline incision was made in the scalp, and a burr hole was drilled at the following coordinates: +0.2 mm anterior and +3.5 mm lateral to the bregma. A 26-gauge Hamilton syringe was inserted to a depth of 5.5 mm from the dura. To cause ICH, we injected 0.32 units of bacterial collagenase type IV (Sigma-Aldrich, St. Louis, MO, USA) dissolved in 1.0 µL of sterile saline over 5 min. The needle was left in place for another 10 min before being slowly pulled out to prevent backflow. A heating pad kept the body temperature at 37 °C during and after surgery.

### 4.5. Hematoma Evacuation and Treatment Implementation

**Figure 7:**
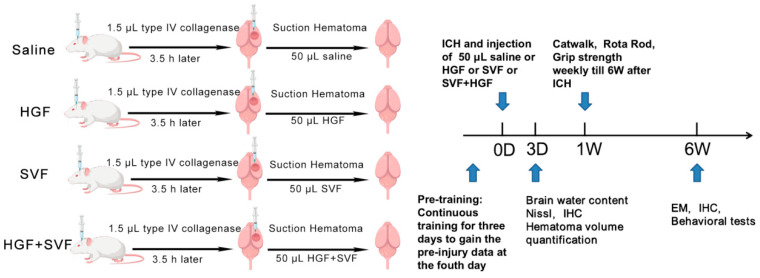
Experimental timeline. Prior to injury, rats underwent pretraining to establish baseline motor performance. At 3 days post-ICH, brain water content, hematoma volume, and inflammatory response were assessed. Behavioral tests (rotarod test, grip strength test and catwalk gait analysis) were conducted weekly until week 6. Electron microscopy was performed at 6 weeks post-injury to visualize the interface between the implanted material and the host tissue.

To better mimic the clinical scenario, a partial hematoma evacuation was done 3.5 h after the ICH induction. The 3.5-h post-ICH treatment window was chosen based on previous studies demonstrating the feasibility of minimally invasive hematoma evacuation within this timeframe [48]. The original burr hole was enlarged, and a blunt 22-gauge cannula connected to a microsyringe pump was put in at the same coordinates. Gentle aspiration was used to remove approximately 30–40% of the hematoma volume based on results from pilot studies. Animals were randomly assigned to four treatment groups right after aspiration. A computer-generated randomization list was used to do this. Each group received a 50 µL intralesional injection through the same cannula: The saline group received sterile 0.9% sodium chloride; the HGF group received 2% HGF; the SVF group received a freshly isolated SVF cell suspension (~2 × 10^6^ cells) in 50 µL saline; and the HGF + SVF group received SVF cells (~2 × 10^6^ cells) thoroughly mixed with 50 µL of the 2% HGF hydrogel just before injection. The cannula was held in place for 5 min after the injection, and the injection rate was set at 2 µL/min. The scalp was then sutured, and the animals were placed in a warm cage to recover (Figure 7). Because the primary focus of acute analyses was to compare therapeutic interventions against the vehicle control, a sham group was not included at the 3-day time point.

### 4.6. Nissl Staining and Quantitative Morphological Analysis of Nissl-Stained Neurons

For morphological assessment of neuronal injury, coronal brain sections (20 µm thickness) were subjected to Nissl staining. Sections were mounted onto gelatin-coated slides and air-dried. They were then hydrated in distilled water for 2 min, followed by staining with 0.1% cresyl violet solution (pre-warmed to 37 °C) for 10–20 min. After staining, sections were rinsed briefly in distilled water to remove excess stain and differentiated in 70% ethanol containing a few drops of glacial acetic acid until the desired contrast was achieved (gray matter clearly distinguishable from white matter). Subsequently, sections were dehydrated through a graded ethanol series (70%, 95%, 100%), cleared in xylene, and coverslipped with neutral mounting medium. Images were captured using a light microscope (Carl Zeiss, Oberkochen, Germany) equipped with a digital camera.

For the morphological assessment of neuronal integrity in the peri-hematomal region, Nissl-stained sections were analyzed using ImageJ 1.53k (NIH, Bethesda, MD, USA). Three to five randomly selected fields per animal (*n* = 6 per group) were captured using a light microscope (Carl Zeiss). Neurons were identified by their characteristic morphology (large cell body, prominent nucleolus, and Nissl substance). The following parameters were quantified: (1) neuronal density, expressed as the number of neurons per mm^2^; (2) mean soma area (μm^2^), measured by manually tracing the cell body of each identified neuron; and (3) circularity index, calculated using the formula 4π × area/perimeter^2^, where a value of 1.0 indicates a perfect circle. All quantifications were performed by an investigator blinded to the experimental groups.

### 4.7. Evaluation of Acute Brain Injury at 3 Days Post-ICH

Measurement of Hematoma Volume: Rats (*n* = 6/group) were anesthetized and transcardially perfused with ice-cold PBS at three days post-injury (dpi). Brains were removed and cut into 1-mm-thick coronal sections with a rat brain matrix. We took digital pictures of each slice. An investigator who was blinded to the groups used ImageJ software (NIH) to measure the hematoma area on each slice three times. The total hematoma volume was then calculated by multiplying the sum of the areas by the slice thickness (1 mm). The volume was given as a percentage of the total volume of the ipsilateral hemisphere.

Brain Water Content (Edema): After slicing the brain, the ipsilateral and contralateral hemispheres were separated and weighed to obtain the wet weight. Then, the tissues were dried in an oven at 105 °C for 72 h to get the dry weight. To determine how much water was in the brain, we used the formula [(wet weight − dry weight)/wet weight] × 100%. For brain water content, one animal from the saline group was excluded due to technical failure during tissue dissection, resulting in *n* = 5 for this outcome. All other analyses maintained *n* = 6 per group.

Acute outcomes were assessed at 3 days post-ICH, while functional recovery was monitored weekly over 6 weeks to distinguish short-term from long-term therapeutic effects.

### 4.8. Histological and Immunofluorescence Examination

At 3 dpi and 6 weeks after the injury, six animals from each group were perfused with PBS and then with 4% paraformaldehyde. After being fixed, the brains were protected from freezing in 30% sucrose and cut into 20 µm-thick sections using a cryostat (Leica, Wetzlar, Germany).

Immunofluorescence staining was performed as follows: sections were blocked with 5% normal donkey serum and incubated overnight at 4 °C with primary antibodies. The following primary antibodies were used: The following primary antibodies were used: NeuN (1:500, Millipore, Burlington, MA, USA), GFAP (1:1000, Abcam, Cambridge, UK), Iba-1 (1:800, Wako, Osaka, Japan), CD68 (1:400, Bio-Rad, Hercules, CA, USA), CD206 (1:500, Abcam, Cambridge, UK), NLRP3 (1:300, AdipoGen, San Diego, CA, USA), cleaved Caspase-1 (1:250, Cell Signaling, Danvers, MA, USA), CD31 (1:300, R&D Systems, Minneapolis, MN, USA), and NF-200 (1:800, Sigma-Aldrich, St. Louis, MO, USA). We used the appropriate Alexa Fluor-conjugated secondary antibodies (1:500, Invitrogen, Carlsbad, CA, USA). 4′,6-diamidino-2-phenylindole (DAPI, Sigma-Aldrich, St. Louis, MO, USA) was used to stain the nuclei. The terminal deoxynucleotidyl transferase dUTP nick end labeling (TUNEL) assay (Beyotime, Shanghai, China) was conducted following the manufacturer’s instructions to identify apoptotic cells.

Image Acquisition and Quantification: Images were acquired using a fluorescence microscope (Carl Zeiss). For each marker, three to five non-overlapping fields were systematically captured from the peri-hematomal region, defined as the area within 500 µm from the hematoma edge in the ipsilateral striatum. Quantification was performed using ImageJ 1.53k by an investigator blinded to group allocation.

For area-based measurements (NLRP3, caspase-1, CD31, NF-200): a fixed threshold was applied after background subtraction, and the positive area was normalized to the total field area.

For cell-based measurements (Iba-1, GFAP, CD68, CD206, NeuN): positive cells were manually counted and expressed as cells/mm^2^.

For TUNEL: the percentage of TUNEL^+^ nuclei among all DAPI^+^ nuclei.

For each marker, all images were acquired using identical exposure settings across all experimental groups to ensure comparability of fluorescence intensities.

### 4.9. Behavioral Functional Assessment

Animals (*n* = 6/group) were tested for behavioral parameters every week for 1 to 6 weeks after the ICH. Behavioral assessments (rotarod, grip strength, CatWalk) were conducted and analyzed by an investigator who remained blinded to the group allocation throughout the entire 6-week experimental period.

Rotarod Test: Using an accelerating rotarod (Ugo Basile, Varese, Italy), we tested motor coordination and balance. The rod sped up from 4 to 40 rpm over the course of 5 min, and rats were placed on it. The latency to fall was recorded, and three trials were performed per session, using the best score for analysis.

Grip Strength Test: A digital grip strength meter (BIO-GS3, Bioseb, Vitrolles, France) was used to measure the strength of the forelimb muscles. The rat was allowed to hold onto a metal grid with its front paws, and it was gently pulled back until it let go. The highest force (in grams) was recorded. Five tests were performed, and the average of the three highest scores was calculated.

CatWalk Gait Analysis: Automated gait analysis was performed using the CatWalk system (Noldus, Wageningen, The Netherlands). Rats were allowed to voluntarily traverse a glass walkway in a dark, non-distracting environment. Trials were considered valid only when the variation in walking speed did not exceed 30% of the mean speed for that animal. A minimum of three valid runs per animal per time point were recorded and averaged.

### 4.10. Electron Microscopy and Analysis of G-Ratio

Six weeks after the injury, rats (*n* = 4/group) were transcardially perfused with a solution of 2.5% glutaraldehyde and 2% paraformaldehyde in 0.1 M PBS. Small tissue blocks (1 mm^3^) from the boundary zone of the lesion/implant site were dissected, post-fixed in 1% osmium tetroxide, dehydrated, and embedded in Epon-Araldite resin. We used uranyl acetate and lead citrate to stain ultrathin sections (70 nm) and examined them with a Hitachi HT-7700 transmission electron microscope. At least 100 randomly selected myelinated axons were analyzed per animal. For EM quantification, the investigator was blinded to group allocation. From each animal, three non-overlapping grid squares from the lesion border zone were systematically sampled. Within each grid, all myelinated axons with clear cross-sectional profiles were photographed, and a minimum of 100 axons per animal were measured. Using ImageJ 1.53k, we measured the G-ratio (axon diameter / total fiber diameter). A lower G-ratio means that the myelin is thicker.

### 4.11. In Vitro 3D Culture of SVF Cells in HGF Hydrogel

To assess SVF cell behavior within the HGF hydrogel, GFP-labeled SVF cells (3.9 × 10^5^ cells/30 μL) were seeded in 2% HGF/350 μL medium and cultured for 8 days. Cell adhesion, proliferation, and morphology were examined by confocal microscopy (LSM700, Zeiss).

### 4.12. Flow Cytometric Immunophenotyping

To characterize the cellular composition of SVF, 5 × 10^5^ freshly isolated SVF cells were placed in flow cytometry tubes. Negative controls (unstained samples) were set up, and antibodies against CD90 PE-Cy7 (Elabscience, Houston, TX, USA), CD31 APC (R&D, Minneapolis, MN, USA), and CD45 APC-Cy7 (Elabscience, Houston, TX, USA) were added to a final volume of 100 μL, followed by incubation at 4 °C for 2 h in the dark. After incubation, 1 mL of PBS was added, and the cells were centrifuged at 300× *g* for 5 min. The supernatant was discarded, and the pellet was resuspended in 200 μL PBS for acquisition. Samples were analyzed using a flow cytometer (D2060R, Aisen Biology (Hangzhou) Co., Ltd., China), and data were processed using FlowJo v10 (BD Biosciences, Ashland, OR, USA).

### 4.13. 10× Single-Cell Transcriptome Sequencing

To gain deeper insight into the cellular heterogeneity of SVF, freshly isolated SVF cells were subjected to 10× single-cell transcriptome sequencing. After quality control, data normalization, and clustering analysis, distinct cell subpopulations were identified based on t-SNE dimensionality reduction and annotated according to canonical marker genes (see Supplementary Methods for detailed protocols).

### 4.14. Statistical Analysis

All results were assessed in a blinded manner. Each experiment was independently repeated at least three times. Normality of data distribution for each outcome was assessed using the Shapiro–Wilk test. Data with normal distributions are presented as mean ± SD and were analyzed using one-way Analysis of Variance (ANOVA), with post hoc Tukey’s test for ANOVA when significant. Non-normally distributed data are presented as median (interquartile range, IQR) and were analyzed using the Kruskal–Wallis test with Dunn’s post hoc test when appropriate. In this study, all the examined parameters including hematoma volume, brain water content, immunofluorescence intensity, behavioral test and ultrastructural EM metrics data were normally distributed and analyzed parametrically.

For behavioral tests assessed weekly (rotarod, grip strength, CatWalk parameters), two-way repeated measures ANOVA was performed to evaluate the effects of group, time, and their interaction. When a significant interaction was detected, Bonferroni’s post hoc test was applied for between-group comparisons at each time point. All statistical analyses were performed using GraphPad Prism 9.0, and a *p*-value < 0.05 was considered statistically significant.

## Data Availability

The datasets utilized and analyzed in this article can be obtained from the corresponding author upon reasonable request.

## Supplementary Materials

The following supporting information can be downloaded at: https://www.mdpi.com/article/10.3390/gels12030257/s1, Figure S1: Complete hemostasis in a rat brain blood vessel; Figure S2: Flow cytometric analysis of SVF cells for CD90, CD31, and CD45. (A) CD31^+^ cells; (B) CD45^+^ cells; (C) CD90^+^ cells; Figure S3: 10× single-cell transcriptome cell subpopulation classification. t-SNE was used to visualize the identified cell subpopulations. Each dot represents a single cell, and distinct colors indicate different cell subpopulations; Table S1: Proportion of different cell subpopulations in rat SVF identified by single-cell RNA sequencing; Figure S4: In vitro 3D culture of SVF cells within the HGF hydrogel.

## Abbreviations

The following abbreviations are used in this manuscript:

ICH	Intracerebral hemorrhage
SVF	stromal vascular fraction
HGF	adhesive self-assembling peptide (HGF-RADA16-IKVAV) nanohydrogel
MSCs	Mesenchymal stem/stromal cells
GFP	green fluorescent protein
SD	Sprague Dawley
dpi	days post-injury
IQR	interquartile range
TUNEL	terminal deoxynucleotidyl transferase dUTP nick end labeling
DAPI	4′,6-diamidino-2-phenylindole
ANOVA	Analysis of Variance
