# Supplementary material for: Combined Stromal Vascular Fraction and HGF-Functionalized Self-Assembling Peptide Hydrogel Improves Intracerebral Hemorrhage Repair in Rats
Source: Gels. 2026 Mar 19;12(3):257. doi: 10.3390/gels12030257 (PMC13026071; doi:10.3390/gels12030257)
Supplement: Supplementary file 1 [file gels-12-00257-s001.zip › gels-4158835-supplementary.pdf]

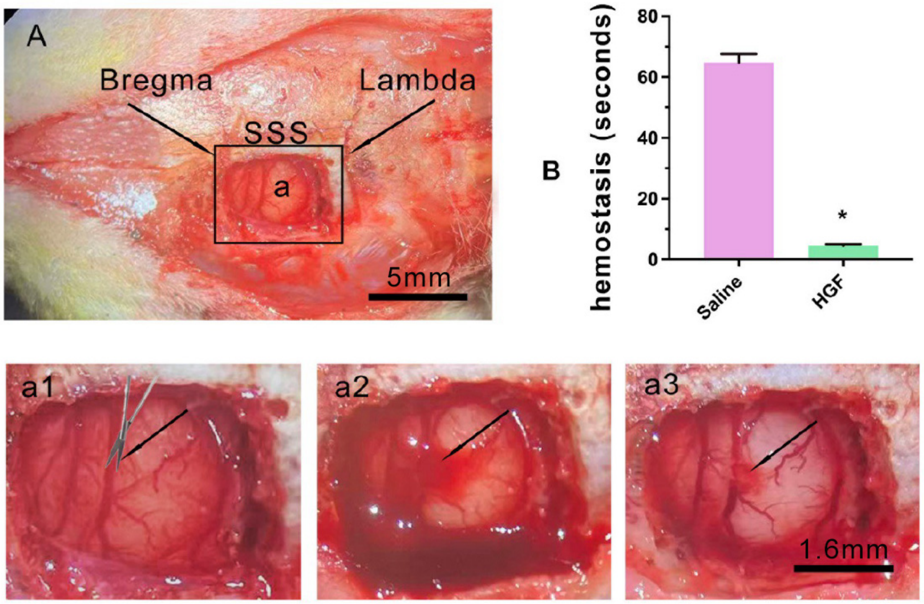

Figure S1. Complete hemostasis in a rat brain blood vessel. A, Image of adult rat brain blood vessels with part of the overlying skull removed. SSS: superior sagittal sinus. The inset "a" in A is magnified in a1-a3. These are time-lapse pictures of each step of the experiment (a1-a3). a1: One of the veins close to the superior sagittal sinus is cut (arrow). a2: The vein that broke open is bleeding (arrow). a3: The same area, seen through the clear HGF, about 4 s after putting 2% HGF on the cut (arrow). Note that the area covered by HGF exhibits full hemostasis. B, The comparison between the saline-treated controls and the HGF-treated group reveals a significant difference. * $p < 0.01$ using Student's t-test. Scale bar in A = 5 mm. Scale bar in a3 = 1.6 mm.

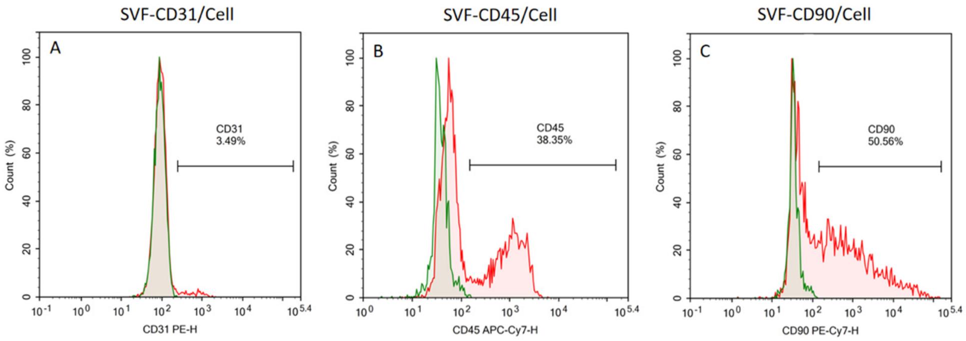

Figure S2. Flow cytometric analysis of SVF cells for CD90, CD31, and CD45. (A) $CD31^+$ cells; (B) $CD45^+$ cells; (C) $CD90^+$ cells.

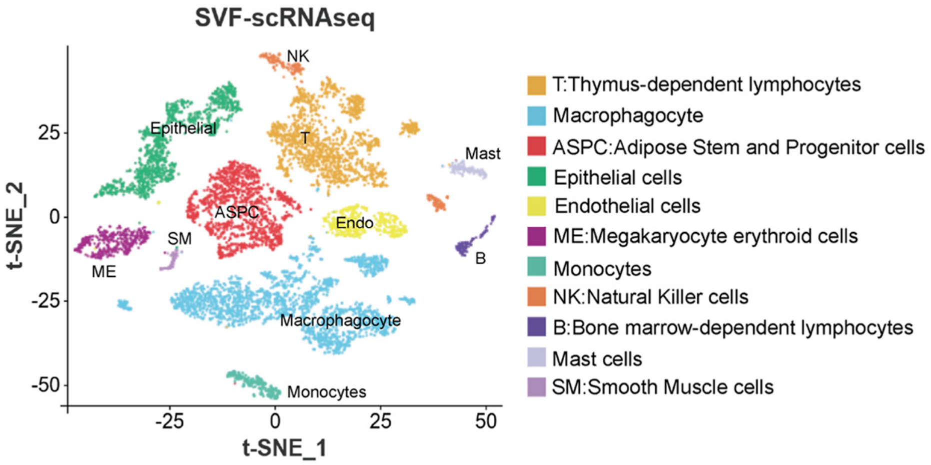

Figure S3. 10X single-cell transcriptome cell subpopulation classification. t-SNE was used to visualize the identified cell subpopulations. Each dot represents a single cell, and distinct colors indicate different cell subpopulations.

Table S1. Proportion of different cell subpopulations in rat SVF identified by single-cell RNA sequencing.

Cluster	Rat SVF Total	Percentage of cells (%)
T	1845	20.01
Macrophagocyte	2583	28.01
ASPC	1544	16.74
Epithelial	1263	13.7
Endo	545	5.91
Megakaryocyte_erythroid	470	5.1
Monocytes	257	2.79
NK	287	3.11
B	167	1.81
Mast	162	1.76
Smooth_Muscle	99	1.07

Notes：SVF: stromal vascular fraction; T: thymus-dependent lymphocytes; Macrophagocyte: macrophages; ASPC: adipose stem and progenitor cells; Epithelial: epithelial cells; Endo: endothelial cells; Megakaryocyte-erythroid: megakaryocyte-erythroid lineage cells; NK: natural killer cells; B cells: bone marrow-dependent lymphocytes; Mast: mast cells; Smooth Muscle: smooth muscle cells. Percentage indicates the proportion of each subpopulation among all 9,222 cells analyzed by single-cell RNA sequencing.

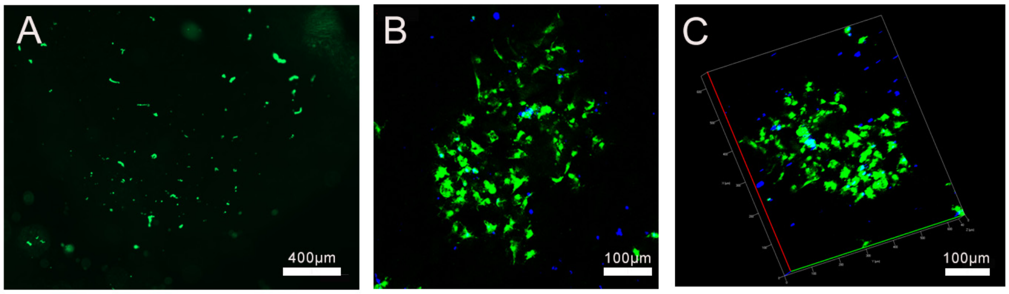

Figure S4. In vitro 3D culture of SVF cells within the HGF hydrogel. (A) GFP-labeled SVF cells at day 0 showing uniform distribution within the hydrogel. (B, C) Confocal microscopy images of

SVF cells on day 8, demonstrating cell adhesion, proliferation, and morphological integrity. Scale bar in A = 200 μm. Scale bar in B, C = 50 μm.
